# *Trichoderma longibrachiatum* (TG1) Enhances Wheat Seedlings Tolerance to Salt Stress and Resistance to *Fusarium pseudograminearum*

**DOI:** 10.3389/fpls.2021.741231

**Published:** 2021-11-16

**Authors:** Solomon Boamah, Shuwu Zhang, Bingliang Xu, Tong Li, Alejandro Calderón-Urrea

**Affiliations:** ^1^Gansu Provincial Key Laboratory of Arid Land Crop Science, Gansu Agricultural University, Lanzhou, China; ^2^College of Plant Protection, Lanzhou, China; ^3^Biocontrol Engineering Laboratory of Crop Diseases and Pests of Gansu Province, Lanzhou, China

**Keywords:** biocontrol microbes, salinity stress, *Fusarium*, *Trichoderma*, mycoparasitism, antioxidative defense system

## Abstract

Salinity is abiotic stress that inhibits seed germination and suppresses plant growth and root development in a dose-dependent manner. *Fusarium pseudograminearum* (Fg) is a plant pathogen that causes wheat crown rot. Chemical control methods against Fg are toxic to the environment and resistance has been observed in wheat crops. Therefore, an alternative approach is needed to manage this devastating disease and the effects of salinity. Our research focused on the mycoparasitic mechanisms of *Trichoderma longibrachiatum* (TG1) on Fg and the induction of defenses in wheat seedlings under salt and Fg stress at physiological, biochemical and molecular levels. The average inhibition rate of TG1 against Fg was 33.86%, 36.32%, 44.59%, and 46.62%, respectively, in the four NaCl treatments (0, 50, 100, and 150 mM). The mycoparasitic mechanisms of TG1 against Fg were coiling, penetration, and wrapping of Fg hyphae. In response to inoculation of TG1 with Fg, significant upregulation of cell wall degrading enzymes (CWDEs) was observed. The expression of β-1, 6-glucan synthase (PP4), endochitinase precursor (PH-1), and chitinase (chi18-15) increased by 1. 6, 1. 9, and 1.3-fold on day 14 compared with day 3. Wheat seedlings with combined TG1 + Fg treatments under different NaCl stress levels decreased disease index by an average of 51.89%; increased the superoxide dismutase (SOD), peroxidase (POD), and catalase (CAT) activity by an average of 38%, 61%, and 24.96%, respectively; and decreased malondialdehyde (MDA) and hydrogen peroxide (H_2_O_2_) content by an average of 44.07% and 41.75% respectively, compared with Fg treated seedlings. The combined TG1 + Fg treatment induced the transcription level of plant defense-related genes resulting in an increase in tyrosin-protein kinase (PR2), chitinase class I (CHIA1), and pathogenesis-related protein (PR1-2) by an average of 1.15, 1.35, and 1.37-fold, respectively compared to Fg treatment. However, the expression levels of phenylalanine ammonia-lyase (PAL) increased 3.40-fold under various NaCl stresses. Our results suggest that TG1 enhances wheat seedling growth and controls wheat crown rot disease by strengthening the plant defense system and upregulating the expression of pathogenesis-related genes under both Fg and salt stress.

## Introduction

Wheat (*Triticum aestivum* L.) is one of the most important cereals grown in arid and semi-arid regions, providing 20% of the total dietary calorie and proteins intake that promotes human nutrition and healthy living (Shiferaw et al., 2013). Drought, soil nutrient deficiency, salinity stress, and disease-causing phytopathogens are all factors that limit wheat development. *Fusarium pseudograminearum* is one of the wheat pathogens that cause catastrophic yield losses by causing crown and root rots in wheat and barley crops (Pasquali et al., 2016). Infected seedlings can experience pre-emergence and post-emergence damping-off as a result of this pathogen (Kazan and Gardiner, 2018). Plants that survive damping-off may exhibit stunted growth and unfilled kernels due to blockage of water and nutrient transfer in the plants. Due to the rot, infected seedlings have dark brown to black crowns. *Fusarium* infections, including *Fusarium* foot rot (FFR) and *Fusarium* root rot (FRR), are responsible for outbreaks of seedling blight, epidemic scab, and *Fusarium* head blight (FHB) in the United States (Subedi et al., 2007). They directly or indirectly cause millions of dollars of losses in wheat and barley production (El-Allaf et al., 2001). The effects of *F. pseudograminearum* on most cereals are often due to the accumulation of deoxynivalenol (DON), an internationally regulated mycotoxin (Bolanos-Carriel et al., 2020). One of the most important methods for controlling plant diseases is chemical control. However, fungicide control of *F. pseudograminearum* in wheat is minimal and does not provide year-round protection against the pathogen (Moya-Elizondo and Jacobsen, 2016; Alahmad et al., 2018) and may pose a risk to human health. Therefore, an alternative for the use of environmentally-friendly and plant-growth-promoting fungi is urgently needed in recent years. Plant-growth-promoting microbes (PGPM) have several potentials that enable them to enhance plant growth. These include ability to fix atmospheric nitrogen and dissolve phosphate in soil, production of siderophores for iron extraction, ACC deaminase that contributes to stress tolerance, and production of indole-3-acetic acid (IAA). They also indirectly promote plant growth by the combating pathogens through nutrient and space competition, secretion of antibiotic substances and induction of defense systems in the plant.

Soil salinization is considered one of the major threats to agricultural production (Isayenkov and Maathuis, 2019), and affects over 1 billion hectares of arable land worldwide (Ivushkin et al., 2019). Increasing salinization is expected to affect 50% of arable land by 2050 (Wang et al., 2003). Salt stress suppresses plant growth, impedes germination and root development in a dose-dependent manner by blocking auxin signaling (Contreras-Cornejo et al., 2014) and triggering dehydration, nutrient deficiency, membrane dysfunction, and oxidative stress, leading to tissue damage or early senescence (Wu and Wang, 2012; Hossain et al., 2017). The accumulation of reactive oxygen species (ROS) is a well-known consequence of stress (Saghafi et al., 2018). Plants evolve scavenging mechanisms that include both enzymatic and non-enzymatic antioxidants to effectively mitigate ROS damage. The major enzymatic systems for ROS scavenging mechanisms, such as SOD, POD, and CAT are important parameters for assessing plant stress resistance. These ROS scavenging mechanisms mediated by antioxidant enzymes are the first line of defense against stress and directly reflect the effects of stress on plants. To maintain the balance between ROS development and interception and to mitigate the negative effects of stress on physiological metabolism and growth of plants, effective antioxidant capacity is essential (Saghafi et al., 2018). Plant responses to combined biotic and abiotic stresses are complex due to the multiple interactions between plants, pathogens and abiotic stresses. It is well known that the physiological and molecular responses of plants to combined abiotic-pathogen stresses differ significantly from their responses to individual stresses (Lin et al., 2015; Ramegowda and Senthil-Kumar, 2015).

Salicylic acid (SA) plays an important role in regulating plant growth, production, maturation, and defense responses (Miura and Tada, 2014). For instance, SA significantly increased seedling size and mass compared to the untreated (control) when applied exogenously to wheat seedlings (Purcǎrea et al., 2010), and high salt tolerance of wheat seedlings was observed when treated with the SA solution (Cornelia et al., 2011). The application of SA as a soil drench stimulates antioxidant protective responses that may lead to systemic acquired resistance (SAR) resulting in *F. pseudograminearum* (Sorahinobar et al., 2016). These findings shed light on the physiological and molecular function of SA in plant resistance to hemibiotrophic pathogens. Therefore, an alternative method of using plant-growth-promoting fungi that can increase SA in plants instead of exogenous SA application is more beneficial to the current agricultural system.

*Trichoderma* species are plant-growth-promoting fungi that play an important role in alleviating abiotic and biotic stresses due to their antimicrobial, mycoparasitic, competitive and secondary metabolic potentials, antioxidant enzyme activity and gene expression and synthesis of phytohormones such as IAA and ACC-deaminase (Qiu et al., 2012; Fu et al., 2017; Rubio et al., 2017). Previous studies reported by Pereira et al. (2014) showed that *T. harzianum* ALL-42 enhanced the response of field bean to *Rhizoctonia solani* by increasing the expression of β-1–3-glucanase and peroxidase genes compared to the host response when exposed to the pathogen alone. Similarly, cucumber roots colonized by *T. harzianum* T-203 showed increased activity of chitinase, β-1,3-glucanase, cellulase and peroxidase at 72 h after inoculation (Chatterton, 2010) which also supports the molecular mechanism of biocontrol- fungi in response to biotic stress.

However, there are fewer reports on the application of *Trichoderma* as biocontrol agent (BCA) in controlling wheat crown rot caused by *F. pseudograminearum* (Fg) and inducing salinity tolerance in wheat seedlings. Thus, the objectives of the present study were (i) to clarify the mycoparasitic mechanisms of TG1 against Fg by morphological and molecular techniques, (ii) to determine the mechanisms of TG1 in controlling wheat crown rot disease under salinity and Fg stress.

## Materials and Methods

### Fungal Inoculum Preparation

The salt tolerance strain of TG1 and Fg were obtained from the Laboratory of Plant Pathology, Gansu Agricultural University. TG1 and Fg were cultured on potato dextrose agar (PDA) in Petri dishes for 7 and 14 days at 25°C, respectively. The conidia suspensions of TG1 and Fg were prepared according to the method of Zhang et al. (2014). The conidial suspensions of TG1 (1.0 × 10^8^ spores per ml) and Fg (5 × 10^8^ spores per ml) were quantified and stored at 4°C.

### Salt Concentration Preparation

Salt tolerance was tested according to Zhang S. et al. (2016) where one liter of liquid medium PDA was prepared and NaCl concentrations of 0, 50, 100, and 150 mM (0, 2.922, 5.844, and 8.766 g) were added and autoclaved at 121°C. The solutions were then poured into 8.5 cm Petri dishes.

### Determination of the Activity of TG1 Against *F. pseudograminearum* Under Salinity Stress

The antagonistic activity of TG1 against *F. pseudograminearum* was tested *in vitro* with modified settings using a dual culture approach by Rahman et al. (2009). The salinity tolerance test was performed using a colony diameter growth approach in PDA media supplemented with 0–150 mM NaCl. In the dual culture approach, mycelial plugs (5 mm diameter) of Fg (7-day-old culture) and TG1 (3-day-old culture) were transferred in parallel to opposite sides of a PDA plate at a distance (1.5 cm) from the outer edge of the plate. The experimental design was completely randomized with two controls, positive control (medium inoculated with Fg and salt), negative control (medium inoculated with Fg without salt), and treatment (medium inoculated with TG1, Fg, and salt). Each of these groups consisted of six replicates. Plates were incubated after inoculation at 25 ± 1°C with intermittent light. Fg colony diameters and percentage of Fg colony growth inhibition (CGI) were determined from the first to the fourth day after incubation and calculated. CGI (%) = [(*C* − *T*)/(*C* − 5)] × 100, where *C* = growth of colony in control, *T* = growth of diameter in treatment and the 5 are the mycelial plugs.

### Observation of the Mycoparasitic Effect of TG1 on *F. pseudograminearum* Using Microscope

The physical interactions between TG1 and *F. pseudograminearum* were monitored using a dual plate assay similar to that mentioned above at 150 mM salt stress. Cellophane fragments were removed from the interaction zone after contact (overgrowth), according to the protocols by Yassin et al. (2019). Using S-3400N Fully Automated VP Scanning Electron Microscope (Hitachi High Technologies America, Inc.) at Gansu Agricultural University, Lanzhou, China, imaging of the <2 mm of the interaction zone was achieved. Samples were dehydrated in 100% ethanol for 1 h, dried with carbon dioxide using a samdri^®^-790 critical point dryer (Tousimis, Rockville, MD, United States), and mounted in aluminum stubs with double-sided tape. The samples were then coated with a tiny layer of gold to make the surface electrically conductive using a Denton DESK II (JMB-3500VA). The electron scans showed the surface structure of the material. The beam settings for surface analysis were 5.0 kV and 1.5 nA, with a spot size of 150. For light microscopy, a wet-mounted glass slide was prepared and viewed under an inverted microscope (Accu Scope, Commack, NY) EXI-410.

### Assessment of TG1 Mycoparasitism Genes

A dual plate assay was performed between TG1 and *F. pseudograminearum*. In a Petri plate containing PDA media with salt (150 mM salt stress) and covered with parafilm, 5 mm mycelial plugs of the pathogen and TG1 were equally spaced following the approach of Lopes et al. (2012). The plates were incubated in the dark at 25 ± 1°C. TG1 mycelia were harvested at three-time points: before contact with *F. pseudograminearum* mycelia (T1), on contact (T2), and after contact (overgrowth) (T3), as defined (Atanasova et al., 2013). The control consisted of TG1 facing itself in a salt medium. Following the method of Pimentel et al. (2020), the induction of mycoparasitism-related genes in TG1 was investigated by quantitative RT-PCR. The selected genes were β-1, 6-glucan synthase (PP4), endochitinase precursor (PH-1), and chitinase (chi18-15). The α-tubulin gene was used as a reference gene. The primers used in the experiments were designed according to the NCBI candidate protein sequences of *Trichoderma* EST. The primer sequences and NCBI genes ID are listed in the Supplementary Table 1. Total ribonucleic acid (RNA) was extracted from the collected samples according to the instructions of the manufacturer of the fungal RNA kit (OMEGA Bio-Tek). The quantity and purity of the isolated RNA were analyzed using a Nanophotometer^TM^ (IMPLEN, Schatzbogen, Germany). The *A*_260_/*A*_28__0_ ratio indicated that the RNA was free from protein contamination. First-strand cDNA (Tiangen Biotechnology, Beijing, China) was synthesized using Revert Aid^TM^ First Strand cDNA Synthesis Kit. qRT-PCR was performed using 2× M5 HiPer Real-time PCR Supermix with Low Rox (Mei5 Biotechnology, Co., Ltd., Beijing, China). According to the manufacturer’s instructions, the 20 μl reaction mixture contained 0.5 μl of each primer, 1.0 μl of cDNA, 10 μl of 2× M5 HiPer Realtime PCR Super mix with Low Rox and 8.0 μl of ddH_2_O qRT-PCR was performed using the following thermal profile: 95°C for 30 s, 40 cycles of 95°C for 15 s, 65°C for 15 s, and 72°C for 30 s. Three technical replicates were used for each gene. The 2^–ΔΔCt^ method (Livak and Schmittgen, 2001) was used to measure the relative expression of each target gene.

### Plant Material and Treatment Conditions

The wheat (*Triticum aestivum* L.) cultivar ‘Yongliang 4’ provided by Gansu Academy of Agricultural Sciences (Lanzhou, China) was used in all the experiments. The cultivar has been well adapted to the major wheat-growing areas of northwest China. The biocontrol potential of TG1 against *F. pseudograminearum* inoculum was tested in artificial saline soil according to the method of Gond et al. (2015). Wheat seeds of equal sizes were surface sterilized with 1% (v/v) NaOCl for 5 min and were rinsed with sterile water six to ten times after disinfection. Thereafter, wheat seeds were soaked in (i) TG1 suspension (1.0 × 10^8^ spores per ml) only, (ii) TG1 suspension plus Fg suspension (5 × 10^8^ spores per ml), (iii) Fg suspension only, and (iv) sterile water only for 12 h. Seeds were air-dried overnight under aseptic conditions before sowing, according to Zhang et al. (2019b).

### Effect of TG1 on Wheat Seeds Emergence Under *F. pseudograminearum* and Salt Stress

Fungi-treated wheat seeds and control seeds were exposed to artificial saline soil at 0, 50, 100, and 150 mM NaCl. The soil used for the study was collected from an agricultural crop field in Lanzhou, China (36.061°N, 103.834°E, and 1,518 m above sea level) had a sandy loam texture. Then completely randomized experimental design with two factorial arrangements having inoculation of TG1 and Fg (plus or minus) as the main variable and salinity as the second variable. For each procedure, 6 plastic pots were used (9 cm in diameter and 10.5 cm in depth), each containing 1 kg of air-dried sterile saline soil. Twenty uniform seeds were sown 1 cm deep in the soil and lightly covered. Plants were irrigated every 24 h with non-saline water and maintained at a constant temperature of 25°C ± 0.5, with supplemental day/night lighting of 16/8 h and relative humidity of 65%. Seedling emergence was counted according to the method Oluwaranti et al. (2015) and the percentage of seed emergence potential (EP%) was determined as follows:


EP(%)=[(seedlingsemergedafter3days)/seedplanted]×100

Based on the formulation of Niu et al. (2013) with minor modification, emergence index [EI (%) = NESi/Ti × 100], where NESi is the number of emerged seeds in a given time and Ti is the incubation time, and emergence rate [ER (%) = (NSE/TNS) × 100], where NSE is the number of emerged seeds 5 days after sowing and TNS is the total number of seeds in each pot.

### Growth Parameters

After 28 days of NaCl treatment, the wheat seedlings were harvested. The shoots and roots of wheat seedlings were removed, washed three times with distilled water, dried, and weighed. Root length and weight were measured with the meter rule and weighing balance. To determine the dry weight, all samples of wheat shoots and roots were oven-dried at 105°C for 30 min and then held at 80°C to maintain a constant weight before being weighed. Each preservation and control was performed six times. The relative water content (RWC) of the shoots and roots was measured according to Tian and Philpot (2015). RWC (%) = [(FW − DW)/FW] × 100, where RWC represents relative water content, FW represents fresh weight, and DW represents dry weight.

### Disease Assessment

Wheat crown rot disease index was recorded 28 days after sowing. A disease index (DI) based on crown rot, yellowing, and chlorosis of cotyledons and leaves at 28 days was used to classify disease symptoms. The degree of the disease was graded using one of five scales adapted from Zhang et al. (2015) where; 0 indicates that there is no disease; 1 = trace to 10% discoloration of the first leaf sheath; 2 = 11 percent −25 percent discolored first leaf sheath; 3 = 26 percent −50 percent discolored first leaf sheath; 4 = 50 percent discolored first leaf sheath or necrotic second leaf sheath; 5 = third leaf sheath necrotic or entire plant badly to entirely rotten.

### Chlorophyll Content Determination

Following the procedure of Miazek and Ledakowicz (2013), chlorophyll was extracted with methanol. A new 0.2 g wheat seedling leaves was homogenized with 10 ml of methanol. At 665 and 652 nm absorbance, the content of chlorophyll was evaluated in a dual-wavelength spectrophotometer (EPOCH2 Plate Reader, BioTek, United States). This was repeated six times.

### Lipid Peroxidation and H_2_O_2_ Measurements

At 28 days after wheat seeds treatments, the root samples were used for oxidants investigations. Oxidants activity such as MDA and H_2_O_2_ were investigated according to the manufacturer’s protocol using the assay kits provided (Solarbio, China). The absorbance of the MDA sample was measured at three different wavelengths 450, 532, and 600 nm, and H_2_O_2_ at 415 nm using a spectrophotometer (EPOCH2 Plate Reader, BioTek, United States). The content of MDA and H_2_O_2_ were expressed as μmol g^–1^ FW. This was repeated six times.

### Antioxidant Enzymes Activities

At 28 days after wheat seeds treatments, the root samples were used for antioxidants investigations. The antioxidants activity of SOD (EC 1.15.1.1), POD (EC 1.11.1.7), PAL (EC 4.3.1.5), and CAT (EC 1.11.1.6) were measured according to the manufacturer’s protocol using the assay kits provided (Solarbio, China). SOD was measured at 560 nm, POD at 470 nm, PAL at 290 nm, and CAT at 240 nm, respectively, using a spectrophotometer (EPOCH2 Plate Reader, BioTek, United States). This was repeated six times.

### Determination of Endogenous SA in Wheat Seedlings

Free SA determination in wheat leaves was performed by high-pressure-liquid chromatography (HPLC) (Shimadzu Prominence LC System, France) following the method of Allasia et al. (2018) with slight modification where 0.1 g of the leaf sample was homogenized using liquid nitrogen. Separations by HPLC were performed on a C18 column (250 × 4.6 mm, 5 μm) using a linear aqueous MeOH gradient from 10 to 82% (v/v), at a flow rate of 1 ml min^−1^, over 30.4 min. Quantify with fluorimetric detection (measured at 305 nm; emission at 407 nm) and determine areas under the corresponding peaks of the standard [2-Methoxybenzoic acid (o-Anisic acid, OAA; internal standard)]. Briefly, stock solution of 152 mg OAA in 10 ml 70% aqueous EtOH (v/v) and diluted 1:1, 000 in ultra-pure water. Using the peak area, the standard curve and linear equation were used to determine the amount of free SA.

### Extraction of Total RNA and Analysis of Gene Expression by Quantitative Real-Time Reverse Transcriptase (qRT-PCR)

Total RNA extraction and analysis of 100 mg wheat seedlings root exposed to different levels of NaCl stress and pathogen infection were performed using the E.Z.N.A.^®^ plant RNA kit (OMEGA Bio-Tek, China) (White et al., 2008; Xie et al., 2013). The quantity and quality of isolated RNA and qRT-PCR were analyzed by the same procedure previously used in the analysis of mycoparasitic gene expression. Following the methodology of Zhang S. et al. (2016), unique primers for the bread wheat genes SOD, POD, and CAT and the internal control actin were used to amplify amplicons specific to wheat seedlings. The above genes were selected based on their role in wheat seedlings under stress in previous studies (Asthir et al., 2011). Primers used in the experiments were designed using Primer Express 3.0 software, which amplifies target genes according to the sequences of candidate proteins available in NCBI wheat EST (Wang et al., 2014). Using the previously described method for mycoparasitic gene expression, the relative expression of antioxidant genes (SOD, POD, and CAT) and the expression level of plant defense-related genes [tyrosine-protein kinase (PR2), PR1-2 pathogenesis-related protein, phenylalanine ammonia-lyase (PAL), and chitinase class I (CHIA1)] were assessed by quantitative qRT-PCR. Primer sequences and NCBI gene IDs for all genes can be found in the Supplementary Table 1.

### Statistical Analysis

The data were tested in each experiment included TG1 strain on controlling *F. pseudograminearum* and inducing salinity tolerance in wheat seedlings. Data were analyzed using two-way ANOVA in SPSS Version 16.0 (SPSS Inc., Chicago, IL, United States), and mean comparisons were made using Duncan’s new multiple range test and the significance was considered at *P* < 0.05.

## Results

### Salinity Tolerance and *in vitro* Colony Growth Inhibition

**FIGURE 1:**
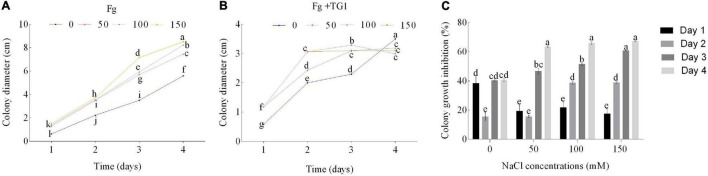
Effect of *T. longibrachiatum* (TG1) and salt stress on *F. pseudograminearum* (Fg) growth. **(A)** colony diameter of Fg only, **(B)** colony diameter of Fg after inoculation with TG1, and **(C)** colony growth inhibition of TG1 against Fg under different salt stresses (0, 50, 100, and 150 mM NaCl) at different days. Different lower case letters indicate significant differences at *P* < 0.05 in Duncan’s multiple range test using two-way ANOVA.

**FIGURE 2:**
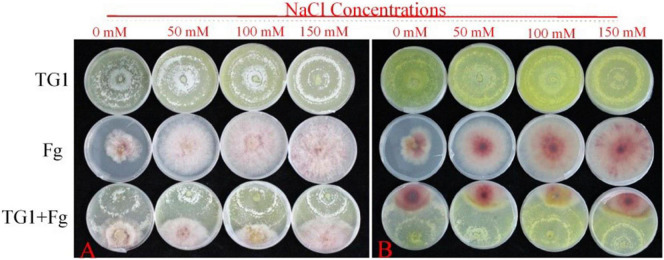
Effect of different NaCl concentrations on colony growth and inhibition between *T. longibrachiatum* (TG1) and *F. pseudograminearum* (Fg) at 4 days after inoculation using dual plate assay (0–150 mM of NaCl concentrations). **(A)** front colony, and **(B)** reverse colony.

Salt stress and TG1 significantly (*p* < 0.05) affected the colony growth of Fg at different days after incubation (Figures 1A,B). The average inhibitory rate of TG1 against Fg was 33.86%, 36.32%, 44.59%, and 46.62%, respectively, for the four NaCl treatments (0, 50, 100, and 150 mM NaCl) (Figure 1C). Additionally, an increase in NaCl concentration increased the inhibitory rate of TG1 against the Fg colony growth at 4 days under different concentrations of salt stress (Figures 1C, 2A,B).

### Microscopic Observations of Mycoparasitism

**FIGURE 3:**
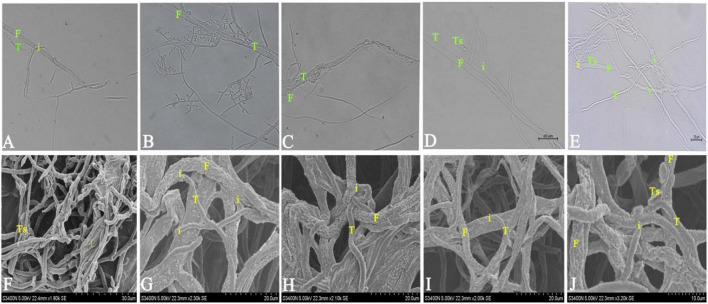
Mycoparasitism of *T. longibrachiatum* (TG1) (T) against *F. pseudograminearum* (Fg) (F) observed under light microscopy at 40× magnification and scanning electron microscopy (SEM) at 4 days after inoculation using dual plate assay at 150 mM NaCl concentration. **(A)** TG1wrapped around *F. pseudograminearum* hyphae with obvious degradation of *F. pseudograminearum* cell wall. **(B)** TG1 conidial growth internally and externally on *F. pseudograminearum* hyphae. **(C)** TG1 hyphal disruption, penetration and breakage of *F. pseudograminearum* hyphae. **(D)** TG1 spore germ tube (Ts) growing on *F. pseudograminearum* hyphae. **(E)** *F. pseudograminearum* hyphae looped by TG1 hyphae and TG1 hyphae extending parallel to *F. pseudograminearum* showing hyphal depression. **(F–H)** SEM images of TG1 (T) coiling the hyphae of *F. pseudograminearum*. **(I,J)** SEM images of TG1 coiling and invading the hyphae of *F. pseudograminearum.* Ts represent *Trichoderma* spore and (i) represent the point of interaction.

Light microscopy revealed that TG1 wrapped around the host hyphae of Fg and formed appressoria and hook-like structures that allowed firm attachment to the fungal host (Figure 3A). Growth of TG1 conidia was observed in the fungal host indicating the use of the fungal host as a nutrient source (Figures 3B,C). Visible disruption and decomposition of Fg hyphae by TG1 was observed coupled with a parallel growth in close hyphal association, also suggesting mycoparasitism. Germinating spore tubes of TG1 were found in the hyphae of Fg (Figures 3D,E). Again, the strain TG1 showed a mycoparasitic mechanism by invading the hyphae of Fg (Figures 3F–J).

### Mycoparasitism Genes

**FIGURE 4:**
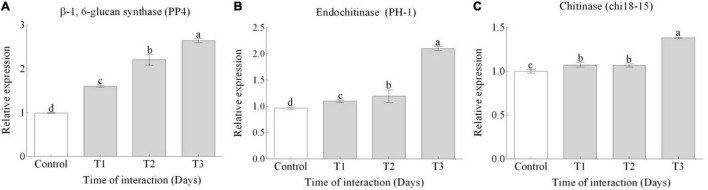
Relative expression levels of **(A)** β-1, 6-glucan synthase (PP4), **(B)** endochitinase (PH-1), and **(C)** endochitinase (chi18-15) genes were measured by qRT-PCR during the interaction of *T. longibrachiatum* (TG1) and *F. pseudograminearum* (Fg) in a dual plate assay at 150 mM salt stress. Gene expression was evaluated at three different interaction time points at Days 3 (T1), 7 (T2), and 14 (T3) after the interaction. The 2^–ΔΔ^*^*c*^*^*t*^ method was used for the relative expression of each target gene using the control and α-tubulin as the reference gene. Small bars represent the standard errors of the means. Different lower case letters indicate significant differences at *P* < 0.05 in Duncan’s multiple range test using two-way ANOVA.

In response to inoculation of TG1 with and Fg, significant (*p* < 0.05) upregulation of CWDEs was observed in TG1 at 150 mM salt stress. The expression of β-1, 6-glucan synthase (PP4) (Figure 4A), endochitinase precursor (PH-1) (Figure 4B), and chitinase (chi18-15) (Figure 4C) genes increased by 1.6, 1.9, and 1.3-fold, respectively, at T3 compared to T1. In appressoria and fast-growing biotrophic hyphae, CWDEs are required for cell wall stiffness, and their rigorous upregulation during biocontrol activity represents a strategy to enhance immunity and mycoparasitism.

### Effect of TG1 on Wheat Seed Growth Under *F. pseudograminearum* and Different Salt Stresses

**FIGURE 5:**
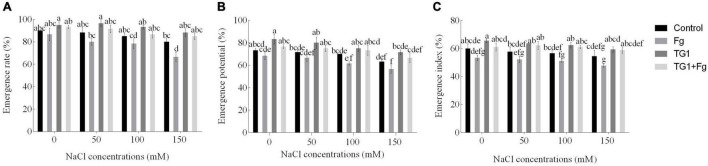
Effect of *T. longibrachiatum* (TG1) on wheat seeds **(A)** emergence rate, **(B)** emergence potential, and **(C)** emergence index under *F. pseudograminearum* (Fg) and salt stress. Different lower case letters indicate significant differences at *P* < 0.05 in Duncan’s multiple range test using two-way ANOVA. Emergence rate, potential, and index were determined after treatment, respectively. Control treatments represent wheat seedlings pretreated with distilled water only; TG1 represents wheat seeds pretreated with the suspension of *T. longibrachiatum* (TG1); TG1 + Fg represents wheat seeds pretreated with *T. longibrachiatum* (TG1) and *F. pseudograminearum* (Fg); and Fg represents wheat seeds pretreated with *F. pseudograminearum* (Fg) for 12 h before planting with different NaCl treatments (0, 50, 100, and 150 mM).

Compared to the treatments with sterile water (control), the seeds treated with TG1 increased emergence rate (ER) (Figure 5A) by an average of 8.71%, emergence potential (EP) (Figure 5B) by 11.39%, and emergence index (EI) (Figure 5C) by 13.19%, in the four NaCl treatments (0, 50, 100, and 150 mM). However, seeds treated with the combination TG1 + Fg increased ER by an average of 15.10%, EP by 15.32%, and EI by 19.15% in all four NaCl treatments compared to the Fg treatment alone. Without TG1 treatment, ER, EP, and EI of the control decreased significantly with increasing NaCl concentration.

### Plant Biomass Accumulations and Relative Water Content

Salt and pathogenic stress significantly (*p* < 0.05) affected fresh weight (FW), dry weight (DW), and relative water content (RWC) of shoot and root of wheat seedlings (Table 1). Compared with the control, TG1-treated seedlings increased FW and DW of the shoot by an average of 36.53% and 20%; the root FW, DW, and RWC by 25.59%, 6.35%, and 11.16%, respectively, across the four NaCl treatments levels (from 0 to 50, 100, and 150 mM). However, TG1 + Fg-treated seedlings increased FW and DW of the shoot by an average of 63.37% and 43.47%; the root FW, DW, and RWC by 56.20%, 35.89%, and 12.05%, respectively, across the four NaCl treatment levels, compared with Fg treatment alone. Increasing NaCl concentration along with pathogen infection without treatment with strain TG1 decreased seedling FW, DW, and RWC.

**TABLE 1:** Effect of *T. longibrachiatum* (TG1) on wheat seedling biomass and relative water content under *F. pseudograminearum* (Fg) and different salt stresses.

** NaCl (mM)**	**Treatment**	**Wheat shoot**			**Wheat root**		
		**Fresh weight**	**Dry weight**	**Relative water**	**Fresh weight**	**Dry weight**	**Relative water**
		**(g plant^–1^)**	**(g plant^–1^)**	**content (%)**	**(g plant^–1^)**	**(g plant^–1^)**	**content (%)**
0	Control	0.600.03bcd	0.100.00c	83.330.24abcd	0.250.04c	0.090.00b	64.001.03abcd
	Fg	0.500.05fg	0.090.00d	82.001.52bcde	0.210.00de	0.080.00c	61.900.05abcd
	TG1	0.760.03a	0.120.01a	84.210.032abcd	0.300.00a	0.100.01a	66.670.03ab
	TG1 + Fg	0.670.02b	0.110.00b	83.580.46abcd	0.280.03b	0.100.00a	64.290.02abc
50	Control	0.580.02cdef	0.100.00c	82.760.44abcde	0.220.06d	0.090.03b	59.091.01cde
	Fg	0.430.03h	0.080.01e	81.401.57cde	0.180.00gh	0.070.00d	61.110.15bcd
	TG1	0.750.00a	0.110.01b	85.330.85a	0.270.00b	0.090.03b	67.670.23a
	TG1 + Fg	0.640.02bc	0.100.01c	84.380.90abc	0.240.03c	0.090.02b	62.500.12abcd
100	Control	0.520.05efg	0.090.00d	82.690.78abcde	0.190.04fg	0.080.00c	57.890.09de
	Fg	0.320.01i	0.060.01f	81.250.69de	0.130.00j	0.060.01e	53.850.32e
	TG1	0.590.02cde	0.090.00d	84.750.89ab	0.240.00c	0.080.02c	66.670.21ab
	TG1 + Fg	0.560.03cdef	0.100.01c	82.141.13bcde	0.220.03d	0.090.01b	59.090.03cde
150	Control	0.300.01i	0.060.00f	80.002.28ef	0.150.00i	0.070.01d	53.330.15e
	Fg	0.230.02j	0.050.00g	78.261.90f	0.090.00k	0.050.00f	44.440.05f
	TG1	0.530.06def	0.090.00d	83.021.38abcde	0.200.00ef	0.080.00c	60.000.01cd
	TG1 + Fg	0.450.01gh	0.080.00e	82.220.30bcde	0.170.03h	0.070.01d	58.820.02cde

*Data are mean ± standard error of replicates in a column followed by different letters are significantly different at *P* < 0.05 based on Duncan’s multiple range test using two-way ANOVA. The treatments are detailed in the footnote of Figure 5.*

### Effect of TG1 on Wheat Seedlings Growth, Chlorophyll Content and ROS Accumulation Under *F. pseudograminearum* (Fg) and Different Salt Stresses

Salt and pathogenic stress significantly (*p* < 0.05) affected shoot height and root length of wheat seedlings (Table 2). Increasing NaCl concentration along with pathogen stress decreased shoot height and root length. However, TG1 treatment increased shoot height and root length of wheat seedlings irrespectively of the presence of the pathogen or different NaCl concentrations (from 0 to 50, 100, and 150 mM). Compared with the control, shoot height and root length increased by an average of 22.76% and 21.35%, respectively, in the seedlings treated with the strain TG1 at the different NaCl concentrations. However, the seedlings treated with TG1 + Fg showed an average increase of 27.21% and 25.21% in shoot height and root length, respectively, at the different NaCl concentrations compared to the seedlings treated with Fg alone.

**TABLE 2:** Effect of *T. longibrachiatum* (TG1) on wheat seedlings growth, chlorophyll content and ROS accumulation under *F. pseudograminearum* (Fg) and different salt stresses.

**NaCl concentration (mM)**	**Treatment**	**Shoot length**	**Root length**	**Chlorophyll content**	**MDA content in root**	**H_2_O_2_ content in root**
		**(cm)**	**(cm)**	**(mg g^–1^)**	**(μmol g^–1^ FW)**	**(μmol g^–1^ FW)**
0	Control	27.13 ± 1.89bc	14.87 ± 0.38bc	1.37 ± 0.11bcd	0.86 ± 0.02ghi	0.99 ± 0.01fg
	Fg	26.10 ± 0.06cd	13.27 ± 1.13def	1.33 ± 0.10bcde	1.01 ± 0.01fg	1.16 ± 0.11f
	TG1	31.83 ± 0.77a	18.03 ± 0.62a	1.72 ± 0.13a	0.70 ± 0.05i	0.55 ± 0.01h
	TG1 + Fg	29.77 ± 0.18ab	15.27 ± 0.49bc	1.63 ± 0.02ab	0.74 ± 0.08hi	0.63 ± 0.10h
50	Control	25.67 ± 0.64cd	14.20 ± 0.06bcde	1.32 ± 0.09bcd	1.10 ± 0.05f	1.17 ± 0.00f
	Fg	25.03 ± 0.33cd	13.17 ± 0.07def	1.26 ± 0.06cdef	1.65 ± 0.05d	1.58 ± 0.04e
	TG1	31.90 ± 1.47a	17.57 ± 0.03a	1.63 ± 0.05ab	0.86 ± 0.01ghi	0.75 ± 0.00gh
	TG1 + Fg	30.87 ± 1.69b	15.13 ± 0.03bc	1.54 ± 0.13abc	0.89 ± 0.01gh	0.84 ± 0.03gh
100	Control	23.97 ± 1.05cde	12.70 ± 0.45efg	1.14 ± 0.07def	1.67 ± 0.06d	1.89 ± 0.04d
	Fg	23.20 ± 0.74de	11.30 ± 0.06g	0.99 ± 0.03f	2.52 ± 0.03b	2.35 ± 0.27c
	TG1	31.73 ± 1.13a	15.57 ± 0.14b	1.51 ± 0.03abc	1.36 ± 0.11e	1.25 ± 0.02f
	TG1 + Fg	30.63 ± 1.62a	13.97 ± 0.27cde	1.36 ± 0.16bcd	1.41 ± 0.08e	1.28 ± 0.02f
150	Control	21.13 ± 1.25e	12.17 ± 0.19fg	1.06 ± 0.05ef	2.21 ± 0.07c	3.82 ± 0.07b
	Fg	16.87 ± 1.49f	8.97 ± 1.02h	0.68 ± 0.06g	4.04 ± 0.02a	5.22 ± 0.03a
	TG1	24.73 ± 0.28cd	14.33 ± 0.22bcd	1.43 ± 0.04abcd	1.34 ± 0.02e	2.11 ± 0.21cd
	TG1 + Fg	23.03 ± 0.23de	13.20 ± 0.25def	1.25 ± 0.14cdef	1.65 ± 0.03d	3.71 ± 0.03b

*Data are mean ± standard error of replicates in a column followed by different letters are significantly different at *P* < 0.05, based on Duncan’s multiple range test using two-way ANOVA. The treatments are detailed in the footnote of Figure 5.*

Compared to seedlings treated with Fg alone, TG1 treatment increased chlorophyll content by an average of 55.39% at the four NaCl stresses. However, the combined TG1 + Fg treatment increased the chlorophyll content of wheat seedlings by 37.14% and 85.38% at 100 and 150 mM NaCl stress, respectively (Table 2).

MDA and H_2_O_2_ content in wheat seedlings treated with control and Fg increased significantly with the increase in NaCl concentration. Averaged over the four NaCl concentration treatments (from 0 to 50, 100, and 150 mM), seedlings treated with strain TG1 decreased MDA and H_2_O_2_ content in roots by 24.28% and 39.79%, respectively, compared to the control; similarly, the combination of TG1 + Fg treated seedlings decreased MDA and H_2_O_2_ content by 44.07% and 41.75%, respectively, compared to seedlings treated with Fg alone. The extent of MDA and H_2_O_2_ content was greater in seedlings treated with Fg at 150 mM NaCl concentration than at 50 and 100 mM NaCl concentrations. Strain TG1 decreased both MDA and H_2_O_2_ content with or without the pathogen and NaCl stress (Table 2).

### Crown Rot, Seedling Blight, and Seedling Death

**FIGURE 6:**
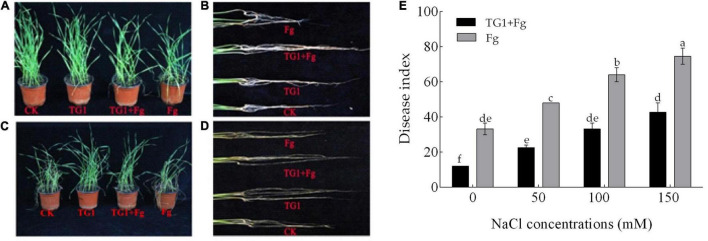
Effect of *T. longibrachiatum* (TG1) on wheat seedling disease index at 28 days after inoculation under *F. pseudograminearum* (Fg) and different NaCl stress. Panels **(A,B)** represent treatments without NaCl; **(C,D)** represent treatments with 150 mM of NaCl stress; and **(E)** represents the disease index across the four NaCl concentrations (0, 50, 100, and 150 mM). Small bars represent the standard errors of the means. Different lower case letters indicate significant differences at *P* < 0.05 in Duncan’s multiple range test using two-way ANOVA. The treatments are detailed in the footnote of Figure 5.

The positive effect of TG1 on reducing the disease index of crown rot was observed when the seeds were treated with BCA. However, the severity of *F. pseudograminearum* increased significantly (*p* < 0.05) in saline soils compared to non-saline soils (Figure 6E). The combined treatment with the antagonist and the pathogen (TG1 + Fg) reduced disease index in wheat seedlings by 64%, 52.78%, 47.92%, and 42.86%, respectively, compared to the pathogen alone across the four NaCl concentrations. The conidiospores of the pathogen germinated and invaded the xylem and pith of both the crown and root, developed a reddish-brown or white discoloration (Figure 6B). Salinity and pathogen stress caused the crown to turn from green to dull yellow, causing dysfunction, and reducing water and nutrient uptake (Figure 6D). Seedling death, root rot and crown rot were observed under pathogen and salinity stress at 150 mM NaCl compared to 0 mM NaCl concentration (Figures 6A,C).

### Endogenous SA Content and PAL Activity

**FIGURE 7:**
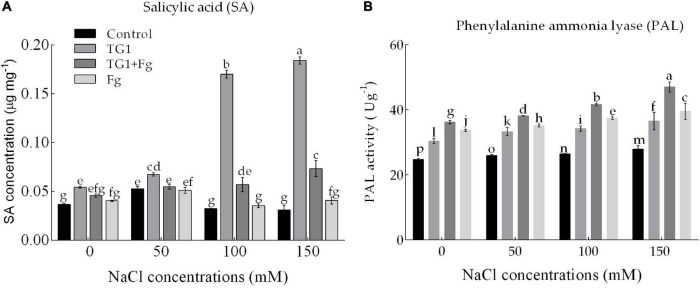
Effect of *T. longibrachiatum* (TG1) on **(A)** salicylic acid content and **(B)** phenylalanine ammonia-lyase (PAL) activity of wheat seedlings subjected to *F. pseudograminearum* (Fg) and different salinity stress at 28 days after inoculation. Small bars represent the standard errors of the means. Different lower case letters indicate significant differences at *P* < 0.05 in Duncan’s multiple range test using two-way ANOVA. Treatments are detailed in the footnote of Figure 5.

NaCl and Fg stress significantly (*p* < 0.05) affected SA content and PAL activity in wheat seedlings (Figures 7A,B). Compared to the control, seeds treated with strain TG1 increased SA by 47.96% at 0 mM NaCl. However, TG1 + Fg treated seedlings increased SA content by an average of 40.56% compared to Fg treated seedlings (Figure 7A).

Fg and NaCl stress significantly affected the activity of PAL. Fg-treated seedlings increased the activity of PAL compared with the control with or without NaCl stress. Averaged over the four levels of NaCl concentration treatment, seeds treated with TG1 + Fg increased the activity of PAL by 11% compared to seedlings treated with Fg alone. Similarly, an increase in NaCl concentration increased the PAL activity of TG1 compared with the control. Across the four NaCl concentration treatments, seedlings treated with TG1 increased the activity of PAL by an average of 28% compared with the control (Figure 7B).

### Antioxidant Enzymes Activities and Gene Expression

**FIGURE 8:**
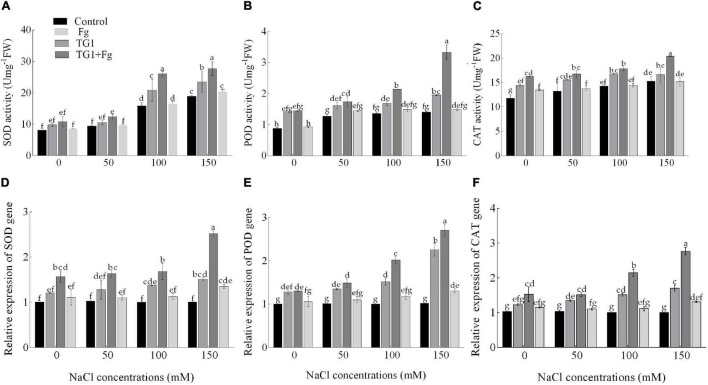
Effect of *T. longibrachiatum* (TG1) on SOD **(A)**, POD **(B)**, and CAT **(C)** activities and relative expressions of SOD **(D)**, POD **(E)**, and CAT **(F)** genes in the root of wheat seedlings under *F. pseudograminearum* (Fg) and different salt stress at 28 days after inoculation. Small bars represent the standard errors of the means. Different lower case letters indicate significant differences at *P* < 0.05 in Duncan’s multiple range test using two-way ANOVA. The treatments are detailed in the footnote of Figure 5.

The combined biotic and abiotic stressors had effects on antioxidant enzyme activity. At all four NaCl concentrations (from 0 to 50, 100, and 150 mM), antioxidant enzyme activity increased with an increasing NaCl concentration. Compared with the control, wheat seedlings treated with TG1 increased SOD activity by 32% and 24% at 100 and 150 mM NaCl concentrations, respectively. However, wheat seedlings treated with TG1 increased POD and CAT at all four NaCl concentrations by an average of 38% and 17%, respectively, compared to the control. Similarly, wheat seedlings treated with the combination TG1 + Fg increased the activity of SOD, POD, and CAT by an average of 38%, 61%, and 24.96%, respectively, compared with Fg treatment alone at the four NaCl concentrations (Figures 8A–C).

Fg and NaCl stress-induced higher transcript levels of the genes SOD, POD, and CAT compared with control. Significant upregulation of the genes SOD, POD, and CAT was observed in response to TG1 treatment (Figures 8D–F). An increase in NaCl concentration along with Fg increased the expression levels of the genes compared with the house-keeping gene. Wheat seedlings treated with TG1 increased the transcript levels of SOD by 1.11 and 1.22-fold at 100 and 150 mM NaCl concentration, respectively, compared with 0 mM NaCl concentration in TG1 treatment. Similarly, the transcript level of POD increased by 1.76-fold in TG1-treated seedlings at 150 mM NaCl concentration compared to 0 mM NaCl concentration. At NaCl concentrations of 50, 100, and 150 mM, TG1-treated seedlings increased the transcription level of the gene CAT by an average of 1.24-fold compared to 0 mM NaCl concentration in TG1 treatment. The combined TG1 + Fg-treated wheat seedlings with 150 mM NaCl-stress increased the expression levels of SOD, POD, and CAT by 1.61, 2.07, and 1.81-fold, respectively, compared to 0 mM NaCl concentration in TG1+Fg treatment.

### Plant Defense-Related Genes Expression

**FIGURE 9:**
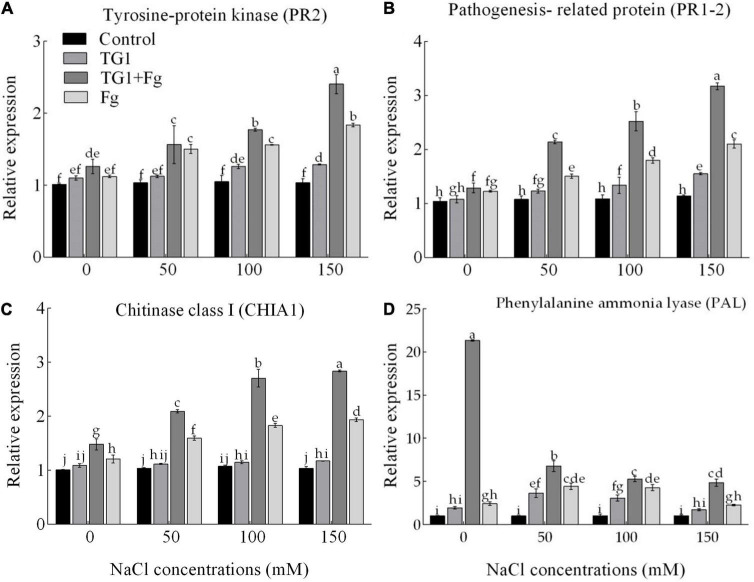
Effect of *T. longibrachiatum* (TG1) on relative expressions of tyrosine-protein kinase PR2 **(A)**, pathogenesis-related protein PR 1-2 **(B)**, chitinase class I CHIA1 **(C)**, and phenylalanine ammonia-lyase **(D)** genes in the root of wheat seedlings under *F. pseudograminearum* (Fg) and different salinity stress at 28 days after inoculation. Small bars represent the standard errors of the means. Different lower case letters indicate significant differences at *P* < 0.05 in Duncan’s multiple range test using two-way ANOVA. Treatments are detailed in the footnote of Figure 5.

NaCl and Fg stress significantly (*p* < 0.05) affected the expression of the proteins associated with pathogenesis (Figures 9A–C). The wheat seedlings treated with strain TG1 increased the transcript levels of the tyrosine-protein kinase (PR2) by 1.15 and 1.17-fold at 100 and 150 mM NaCl concentration, respectively, compared to 0 mM NaCl concentration in TG1 treatment. Similarly, transcription of pathogenesis-related protein (PR1-2) increased by 1.24 and 1.44-fold at 100 and 150 mM of NaCl concentration, respectively, under TG1 treatment. Likewise, the transcript level of chitinase class I (CHIA1) in TG1-treated wheat seedlings increased by 1.08-fold at 150 mM NaCl concentration compared to 0 mM NaCl concentration in TG1 treatment. However, the PAL transcript level of TG1-treated wheat seedlings increased by 1.87 and 1.58-fold at 50 and 100 mM NaCl compared with 0 mM of NaCl concentration, respectively (Figure 9D). The combined TG1 + Fg treatment induced the transcription level of plant defense-related genes resulting in an increase in PR2, CHIA1, PR1-2, and PAL gene expression by an average of 1.15, 1.35, 1.37, and 3.40-fold, respectively, compared to Fg treatment.

## Discussion

High NaCl stress affected colony color and sporulation of both the antagonist and the pathogen. Previous studies found that salt stress decreased colony diameter growth, spore germination, morphology, conidial sporulation and environmental sensitivity of the fungus (Paulitz and Bélanger, 2001). Other studies revealed that *F. solani* was the most resistant among the *Furarium* to an increase in NaCl and temperature (Mandeel, 2006). The NaCl tolerance of the species was followed by the development of massive thick-walled, drought-resistant chlamydospores, indicating that the species is predominantly thermo-osmotolerant (Mandeel, 2006). Similarly, in this study, both the antagonist and the pathogen tolerated 150 mM NaCl stress. *Furarium pseudograminearum* tolerated high sodium chloride stress with slow growth compared to the antagonist. Both fungi developed salt-resistant hyphae, thick-walled conidia and chlamydospores. Interestingly, the antagonist *T. longibrachiatum* TG1 inhibited the growth of the pathogen under both normal and NaCl stress, which is helpful for its activity as a BCA. This result is in agreement with the findings of Narayan et al. (2017) whose study showed that *T. viride* and *Trichoderma* spp. significantly inhibited the mycelial growth of *Fusarium*.

In the present study, the microscopic analysis showed that TG1 wraps around the hyphae of *F. pseudograminearum* and grows parallel with the tight hyphae association, indicating mycoparasitism (Monteiro et al., 2010). Previous studies have shown that during mycoparasitism, *Trichoderma* wraps around the hyphae of the fungal host and forms appressoria and hook-like structures that allow firm attachment to the fungal host (Sachdev and Singh, 2020). Also in this study, during the interaction between *T. longibrachiatum* TG1 and *F. pseudograminearum*, coiling, disintegration of host hyphae, formation of appressoria and hook-like structures and penetration into the hyphae were observed. This suggests that the mechanisms of host recognition and lysis by *Trichoderma* may be different in different *Trichoderma*-host interactions. In addition, a possible CWDEs activity was detected with the degradation of *F. pseudograminearum* cell walls and hyphal deepening in the area infested by *T. longibrachiatum* TG1. These results were confirmed by the findings of Vinale et al. (2008) who reported that CWDEs can degrade or hydrolyze the cell wall of host pathogens and inhibit their growth.

*Trichoderma* spp. are well-known BCA with demonstrated activity against a range of pathogenic fungi (Harman and Uphoff, 2019). CWDEs, such as chitinases and glucanases, have been reported to be directly involved in the mycoparasitic activity of *Trichoderma* by facilitating lysis of the fungal host cell wall during parasitism, thus enabling invasion of the fungal host (Jaroszuk-Ściseł et al., 2019; Pimentel et al., 2020). *Trichoderma longibrachiatum* TG1 showed an increase in the expression level of CWDEs upon interaction with *F. pseudograminearum*. Significant expression of β-1, 6-glucan synthase (PP4), endochitinase (PH-1), and chitinase (chi 18-15) was conspicuous after contact between the two fungi, at a time when *T. longibrachiatum* TG1 overgrew *F. pseudograminearum*. The significant increase in expression of genes during the interaction of TG1 with *F. pseudograminearum* suggests that these enzymes are involved in the mycoparasitic activity of BCA against *F. pseudograminearum*. Previous studies have shown that genomic studies of mycoparasitic *Trichoderma* species have revealed higher numbers of chitinases and glucanases (Karlsson et al., 2017). In other studies, induction and secretion of β-1,3-glucanase and chitinase have been shown to play a role in the mycoparasitism of *F. oxysporum* by *Trichoderma* species (Rashad and Moussa, 2020) and mycoparasitism of *Sclerotinia sclerotiorum* by *T. harzianum* T-aloe (Zhang F. et al., 2016). Consistent with this study, other secondary metabolites produced by *T. longibrachiatum* TG1 species include these CWDEs (Lorito et al., 1994). In biocontrol, these enzymes are often assigned to both parasitism and antibiosis. CWDEs such as chitinase, β-1,3-glucanases, and cellulase, for example, were not only essential features of the mycoparasitism for colonizing the pathogen but also had a significant antifungal effect on the pathogen. This result is in agreement with that of Yuef et al. (2018) where the chitinase and glucanase activities of *T. asperellum*, *T. virens*, *T. gamsii*, and *T. logibrachiatum* inhibited 15% of mycelia growth of *Phytophthora parasitica* and 45% of *F. oxysporum*. The identification of biological control mechanisms that are highly effective against plant pathogens represents a tool that can be explored for plant disease management, especially in the case of important phytopathogens that are difficult to control, such as *F. pseudograminearum*.

In the pot experiment, the different NaCl concentrations and the pathogen decreased the emergence parameters of wheat seeds, which is clearly in accordance with the hypothesis of the study. Several previous studies have reported the adverse effects of salinity on germination and growth of plant seeds both *in vitro* and in the greenhouse (Azooz et al., 2013). Similarly, sodium chloride treatment was reported to inhibit root hair growth (Dey et al., 2004). Previous studies also showed that *F. pseudograminearum* infection increased from less than 20% 10 days after anthesis to more than 98% at maturity under significant environmental stress in both resistant and susceptible wheat cultivars, resulting in unacceptable standard germination and seed vigor (Argyris et al., 2003).

In the present study, the interaction of pathogen and salt stress resulted in poor seed germination and seed vigor. However, the combined treatment (TG1 + Fg) improved seedling emergence and vigor compared with the pathogen alone treatment. Moreover, the application of *T. longibrachiatum* TG1 reduced the deleterious effects of NaCl stress and the pathogen on wheat seedling growth, increasing shoot and root length and enhancing root hair formation, which is beneficial for stressed plants. These results were supported by Mastouri et al. (2012) who reported that application of *T. harzianum* T22 improved germination of tomato (*Lycopersicum esculentum* L.) seeds under abiotic and biotic stress conditions. Similarly, *T. longibrachiatum* T6 promoted the growth of wheat seedlings (*T. aestivum* L.) under NaCl stress by increasing shoot and root length (Zhang et al., 2019b). These results and the general observation show that *Trichoderma* treatments benefit plants most when they are stressed, support the theory that these beneficial fungi alleviate both biotic and abiotic plant stress.

In this study, *T. longibrachiatum* TG1 induced salt tolerance and reduced disease incidence (e.g., seedling blight, crown rot caused by *F. pseudograminearum* on wheat seedlings). In previous studies, *Trichoderma* was shown to promote plant root growth and nutrient solubilization (Contreras-Cornejo et al., 2009). The adverse effects of *Fusarium* wilt are caused by the blockage of xylem vessels leading to leaf senescence and reduced photosynthesis which in turn leads to lower crop yields as previously reported (Singh et al., 2017). The antagonist was an endophytic fungus that increased the relative water content and membrane protection. Infection with a pathogen usually triggers systemic acquired resistance (SAR), a general defense response, first in infected host cells, then in uninfected neighboring tissues. Similarly, the interaction of pathogen and salt stress increased the disease index of wheat seedlings due to the salt tolerance potential of the pathogen. The antagonistic effect of *T. longibrachiatum* TG1 against salinity and the pathogen seems to be related not only to direct competition and mycoparasitism but also to activation of plant defenses through the activation of SA, the synthesis of phytohormones and an increased number of ROS scavenging antioxidant enzymes and the maintenance of osmotic balance and metabolic homeostasis in wheat seedlings. This result was supported by Hasan et al. (2014) who found that the production of lytic enzymes by antagonists has been shown to control pathogenic fungi by competing for tissue nutrients, thus suppressing plant pathogens caused by *Trichoderma* spp.

The abiotic and biotic stresses in this study affected the chlorophyll of the seedlings and decreased the relative water content. Chlorophyll is an important molecule in photosynthesis, and plant bioregulators help to increase the quality of chlorophyll by maintaining cellular osmotic reactions (Burman et al., 2004; Seckin et al., 2009). The decrease in chlorophyll content in wheat under pathogen and salt stress may be due to increased chlorophyll degradation or decreased activity of chlorophyll biosynthesis enzymes (Singh et al., 2016; Mann et al., 2019). Larger decreases in chlorophyll concentration have also been associated with exacerbation of stress symptoms (Kumar et al., 2018). Previous studies have shown that salinity and *F. pseudograminearum* are known to affect photosynthetic processes in most plants by altering organelle ultrastructure, the concentration of various pigments, metabolites and enzymatic activities (Akram et al., 2018). However, under both stress conditions, the antagonist *T. longibrachiatum* TG1 maintained cells at an optimal hydration level by accumulating osmolytes, which maintained water uptake and increased tissue RWC (Yadav et al., 2020). Other studies have also shown that enhancement of gas exchange properties by endogenous SA through the application of *T. longibrachiatum* helps to improve photosynthetic efficiency and chlorophyll fluorescence, which enables plants to withstand environmental stress (Wang and Li, 2005). Osmolytes are stress tolerance systems that plants use to protect themselves from abiotic stress (Li and Dami, 2016). Similar to the current study, chlorophyll content increased when *T. longibrachiatum* TG1 and the pathogen were applied together, with the antagonist causing rapid root colonization due to its rapid growth and high salt tolerance. This was due to the delay of pathogenic stress and promotion of root development for water and nutrient uptake. In seedlings exposed to salt and pathogen stress, RWC in the plant decreased and photosynthetic structures were damaged due to reduced nutrient uptake.

Endogenous SA which enhances osmotic changes serves as a stress indicator that stabilizes macromolecules and protects against the effects of ROS (Ahmad et al., 2012; Lata et al., 2019). In both treatments, higher endogenous SA content correlated with higher stress intensity as shown by the results of this study (Khan et al., 2013). Previous studies have shown that abiotic and biotic stresses trigger the oxidation of acids in the lipid bilayer leading to a shift in the cell membrane (Carrasco-Ríos et al., 2013). In this study, MDA levels increased in wheat seedlings under saline conditions, while pathogen infection doubled the effects. Higher MDA levels were associated with increased electrolyte loss and H_2_O_2_ accumulation (Nandwal et al., 2007), which has long been used as a marker of stress tolerance due to lipid peroxidation. However, *T. longibrachiatum* TG1 induced SA to decrease fatty acid degradation, increase antioxidant enzyme activity and decrease MDA and H_2_O_2_ levels (Abdelkader et al., 2012). Moreover, wheat seedlings treated with the antagonist and pathogen with or without NaCl accumulated low H_2_O_2_ and MDA levels by increasing the activities of scavenging ROS antioxidant enzymes (SOD, POD, and CAT), compared to what was reported by Zhang et al. (2019a) where *T. harzianum* T-soybean increased scavenging antioxidant enzymes for ROS in cucumber under salt stress.

The expression levels of antioxidant genes were upregulated at all stress levels, which stimulated stress response signaling, increased shoot height and root length, and further increased biomass content in the seedlings treated with the antagonist and the pathogen (TG1 + Fg) compared seedlings treated with the pathogen alone and mock seedlings. This result is similar to that of Zhang et al. (2019c) where *T. longibrachiatum* T6 promoted the growth of wheat seedlings under salt stress through an antioxidant defense system. However, this study involves a different BCA strain that combats both salt and pathogen stress through enzymatic antioxidants, expression of protein enzymes associated with pathogenesis and induction of endogenous SA content of seedlings. Previous studies have shown that exogenous SA treatment increases the transcripts of genes encoding ascorbate and glutathione cycle enzymes (Chen et al., 2016), and overexpression of these genes conferred increased resistance to salt and chilling stress. SA, a phenolic phytohormone, regulates plant growth and production, photosynthesis, transpiration, ion absorption, and transport (Khan et al., 2014), and thus SA has shown positive responses in mitigating both pathogenic and osmotic stress (Chini et al., 2004). Similarly, the increase of endogenous SA by TG1 played an important role in the improvement of the root system, which allowed correction of salt-induced growth and improved biomass production by inducing the expression of enzymatic antioxidants and plant protective genes in plants. Our results are in agreement with those of Luan et al. (2020), who showed that *Trichoderma* isolates ThTrx5 confers salt tolerance to *Arabidopsis* by activating stress response signals and overexpressing of SOD, POD, and CAT, and increase root length and fresh weight of ThTrx5 transgenic plants.

Salicylic acid regulates the activities of various enzymes, such as enzymatic antioxidants and PAL, which are the main components of induced plant protection against biotic and abiotic stresses. In this study, the salicylic acid pathway was determined by the activity of PAL. Previous studies reported that PAL plays a central role in the hypersensitive response (HR) and SAR is associated with early signaling events in response to pathogens, such as activation of PR genes (Dong, 2001; Gruner et al., 2013). By catalyzing cell wall lignification, these enzymes play an important role in protecting plants from pathogens. Other studies have shown that rapid SAR-activation through the expression of various PR genes leads to a significant increase in resistance to pathogen infection (Qi et al., 2019). Pretreatment of wheat seedlings with *T. longibrachiatum* TG1 increased endogenous SA-content and stimulated cell wall degrading enzymes and plant cell wall lignification to reduce pathogen infection. Previous studies by Valifard et al. (2015) showed that salt stress could induce PAL expression (12 to 18 times) and subsequently increase PAL activity (42%–45%) and total phenolic accumulation (35%–43%) in the early hours after stress treatment. Again, Cuong et al. (2020) revealed that, treatment with various concentrations of NaCl (50, 100, and 200 mM) resulted in increased epicatechin levels but decreased accumulation of benzoic acid (TaPAL) in wheat sprouts compared with the control (0 mM). Upregulation of PAL and the enzymes PR1, PR1-2, and chitinase reduced the number of certain mycotoxin forms that accumulated in seedlings and induced salt tolerance. The current study results are in agreement with those of Pereira et al. (2014) who reported that *T. harzianum* ALL-42 appears to enhance the response of field beans to *R. solani* by increasing the expression of β-1–3-glucanase (glu1) and peroxidase (pod3) genes, compared to the host response when exposed to the pathogen alone. Similarly, cucumber roots colonized by *T. harzianum* T-203 showed increased activity of chitinase, β-1,3-glucanase, cellulase, and peroxidase 72 h after inoculation (Chatterton, 2010) which also supports the current result. Therefore, another mechanism by which *T. longibrachiatum* TG1 increases seedling salt tolerance and resistance to pathogen attack could be realized via the SAR pathway. These results are supported by those of Ali et al. (2018) who found that genes related to pathogenesis (PR) show basal expression under control conditions but increase dramatically after fungal infection both at the locally infected site and in uninfected parts of the host, triggering the SAR pathway.

## Conclusion

Our results suggest that *T. longibrachiatum* TG1 is a plant growth-promoting fungus that can tolerate salt stress and control *F. pseudograminearum in vitro* and *in vivo*, and induce salinity tolerance of wheat seedlings by attenuating the negative effects of pathogen and salt stress. Rigorous physiological, biochemical, and molecular assays used in the study allowed us to explore the possible mechanisms and pathways in which TG1 attenuates the suppressive effects of pathogen and salt stress. The mechanism involves are; (i) mycoparasitism (ii) overgrowth and space competition (iii) antioxidant defense system and expression and increase of endogenous SA in stressed plants. The identification of biological control agents that are highly effective against salt stress and plant pathogens represents a tool that can be explored for plant disease management, especially in the case of important phytopathogens that are difficult to control, such as *F. pseudograminearum*. These results and the repeated observation show that the greatest benefit of *Trichoderma* treatments to plants occurs when they are under stress, lend credence to the concept that these beneficial fungi alleviate both biotic and abiotic plant stress. Our results provide a basis for future incorporation of biological control agents into strategies to control seedling blight, crown rot, and root rot of plants under salt stress.

## Data Availability Statement

The raw data supporting the conclusions of this article will be made available by the authors, without undue reservation, to any qualified researcher.

## Author Contributions

SB and TL performed the experiments, analyzed data, and wrote and edited the manuscript. SZ, BX, and AC-U conceived the idea and revised the manuscript. SZ designed the experiments, conceived the idea, and revised the manuscript. All authors have read and agreed to the published version of the manuscript.

## Conflict of Interest

The authors declare that the research was conducted in the absence of any commercial or financial relationships that could be construed as a potential conflict of interest.

## Publisher’s Note

All claims expressed in this article are solely those of the authors and do not necessarily represent those of their affiliated organizations, or those of the publisher, the editors and the reviewers. Any product that may be evaluated in this article, or claim that may be made by its manufacturer, is not guaranteed or endorsed by the publisher.

## Abbreviations

IAA: indole-3-acetic acid
ROS: reactive oxygen species
MDA: malondialdehyde
H_2O2_: hydrogen peroxide
PGPR: plant growth-promoting rhizobacteria
RNA: ribonucleic acid
ACC: 1-aminocyclopropane-1-carboxylate
SOD: superoxide dismutase
POD: peroxidase
CAT: catalase
DNA: deoxyribonucleic acid
ER: emergence rate
EI: emergence index
EP: emergence potential
NaCl: sodium chloride
Fg: *Fusarium pseudograminearum*
SEM: scanning electron microscopy.
