# Supplementary material for: Trichoderma longibrachiatum (TG1) Enhances Wheat Seedlings Tolerance to Salt Stress and Resistance to Fusarium pseudograminearum
Source: Front Plant Sci. 2021 Nov 16;12:741231. doi: 10.3389/fpls.2021.741231 (PMC8635049; doi:10.3389/fpls.2021.741231)
Supplement: Supplementary file 1 [file Table_1.docx]

SUPPLEMENTARY MATERIALS

Table 1. Gene description, primers sequences, and NCBI gene ID for the genes used for the qRT-PCR

| **Gene symbol** | **Description** | **Primer sequence (5’ -3’)** | **Gene ID** | **Organism** | **Experiment** |
| --- | --- | --- | --- | --- | --- |
| SOD | Manganese superoxide dismutase | F: GAAGAACCTCAAGCCTATCAGCG  R: CAGAGGGTGCTTTACAAGGATCT | 542833 | *Triticum aestivum* | Antioxidant |
| POD | Peroxidase | F: GCCGTTGAGATTACTGGTGGAC  R: GTCTTCCTGATGCTACCAAGGG | 606365 | *Triticum aestivum* | Antioxidant |
| CAT | Catalase | F: GCTGGGGTCAACACTTACATGC  R: GAGGAAGCTATCAGAGTTGGAGGA | 100682478 | *Triticum aestivum* | Antioxidant |
| ADF7 | actin-depolymerizing factor 7 | F: GCTCCTAGAGCTGTATTCCCAAGT  R: CAGTCGAAACGTGGTATCTTGACT | 101290623 | *Triticum aestivum* | Antioxidant |
| PP4 | β-1,6-glucan synthase | F: CGCCCAAGGACATCCTGATT  R: ACGCTTTATTCGCGGTTTCG | 45525524 | *P. putida* | Mycoparasitism |
| PH-1 | Endochitinase precursor | F: ACTGGGGCTGATTCTCTCCT  R: CCCAACTCCCGATATGCCAA | 29985161 | *T. gamsii* | Mycoparasitism |
| Chi18-15 | Chitinase (Chi18-15) | F: TGTGAAAGTTGCAACGCCAG  F: CCGCCATTGACCTTAGCTCA | 18486528 | *T. reesei* | Mycoparasitism |
| M431 | α-tubulin | F: AAAGGAGGATGCGGCGAACAA  R: AGTGTTGATGAGGCGGCTTGT | 36627008 | *T. harzianum* | Mycoparasitism |
| PR2 | Tyrosine-protein kinase | F: ACTTTTCAGAGGAAGCAGCGA  R: AGTCTGCCGATGGTATACACAA | 107266364 | *Triticum aestivum* | Plant defense |
| PR 1-2 | Pathogenesis-related protein | F: GCAAAGGTGACTGCCAACTG  R: AAGTGGCACAGATGCAGTGA | 543422 | *Triticum aestivum* | Plant defense |
| CHIA 1 | Chitinase I | F: GCCATGTGCTGCTGGTAAAA  R: AGCATTTTAACCCCACCCCC | 542775 | *Triticum aestivum* | Plant defense |
